# Nutrition and Dementia

**DOI:** 10.1155/2012/926082

**Published:** 2012-06-18

**Authors:** Fabio Coppedè, Paolo Bosco, Andrea Fuso, Aron M. Troen

**Affiliations:** ^1^Faculty of Medicine, Section of Medical Genetics and Pisa University Hospital (AOUP), Via S. Giuseppe 22, 56126 Pisa, Italy; ^2^IRCCS, Oasi Maria SS Institute for Research on Mental Retardation and Brain Aging, Via Conte Ruggero 73, 94018 Troina, Italy; ^3^Department of Psychology, Section of Neuroscience, Sapienza University of Rome, Via dei Marsi 78, 00185 Rome, Italy; ^4^Nutrition and Brain Health Laboratory, Institute of Biochemistry, Food Science, and Nutrition, The Robert H. Smith Faculty of Agriculture, Food, and Environment, The Hebrew University of Jerusalem, Rehovot 71600, Israel

Dementia is a progressive decline in the ability to remember, learn, understand, and communicate. Alzheimer's disease (AD) represents the most common form of dementia in the elderly, affecting about 28 million individuals worldwide. Current treatments for AD and other dementias are sorely limited, falling short of preventing or significantly slowing disease progression. Worldwide, the number of people afflicted by AD is expected to exceed 100 million by 2050, as a result of the increased life-span expectancy in both developed and developing countries [1]. Compelling epidemiological observations suggest that human nutrition and lifestyle factors can modify the risk of late onset dementia. For example, overweight in midlife has been associated with an increased risk for dementia in late life [2], and frequent consumption of fruits and vegetables, fish, and omega-3 rich oils is associated with decreased AD risk. Dietary patterns such as the “Mediterranean diet,” characterized by high intake of vegetables, legumes, fruits, cereals, and unsaturated fatty acids have also been associated with decreased risk of developing mild cognitive impairment (MCI) and of MCI conversion to AD [3]. There is also evidence that higher intake and status of specific nutrients such as vitamin E, vitamin B12, and folate may be protective against cognitive decline and aging [4]. Similarly, intake of antioxidants, such as vitamin E and C, and fatty fish have been found to be protective against vascular dementia (VaD) risk, whilst fried fish intake and elevated homocysteine are associated with increased risk [5]. 

Despite this, evidence for the cognitive benefit of nutritional and lifestyle interventions in age-associated cognitive impairment and dementia remains equivocal, and a clear elucidation of mechanisms remains elusive. Although some good evidence is available for the beneficial effect of nutritional interventions on neurocognitive outcomes [6], most large scale nutritional randomized clinical trials to date have failed to clearly demonstrate efficacy in mitigating cognitive impairment and dementia (as well as other chronic conditions). This gap between the epidemiological evidence and interventional trials has prompted a critical re-evaluation of conceptual and practical limitations of clinical trials of nutritional interventions and the need for better trial design [7–9], while at the same time redoubling efforts in fundamental research to clarify the underlying pathophysiological mechanisms of the indicated interventions.

This special issue presents timely review articles and research papers covering several aspects of nutrition in dementia providing a forum for the critical evaluation and delineation of new approaches and opportunities for nutritional and lifestyle interventions. Three papers in this issue address the role of obesity in dementia. R. Businaro et al. reviewed the molecular mechanisms linking obesity to AD risk, focusing on the correlation between the onset and progression of the disease and the stress-induced changes in lifestyle, leading to overnutrition and reduced physical activity, ending with metabolic syndrome and obesity. Particularly, the authors reviewed the factors leading to alterations of energy metabolism in favour of visceral fat accumulation and the subsequent promotion of insulin resistance and chronic inflammation, both critical factors for AD initiation and progression. They also discussed strategies to reduce abdominal fat deposits and their beneficial role on cognitive decline in a comprehensive and updated review article. It is clear from this review that hyperinsulinemia is one of the most frequent endocrine features in overweight people leading to insulin desensitization and represents a risk factor for cognitive decline. This point was discussed also by L. Moll and M. Schubert that reviewed the literature dealing with the role of insulin and insulin-like growth factor-1 in the pathogenesis of obesity-associated dementia, with focus on the possible contribution of forkhead-box transcription factors (FoxO). FoxO are mediators of insulin and insulin-like growth factor-1 involved in several processes including neuronal proliferation, differentiation, stress response, and *β*-amyloid detoxification. In this original review article, the authors discuss the few studies performed so far in animal models to investigate the possible contribution of FoxO-mediated transcription to AD pathology. Studies in *C. elegans* are in conflict with those performed in mice that suggest that FoxO-mediated transcription does not protect against but rather increase amyloid pathology. However, the small number of published papers limits our understanding of the role of this pathway in dementia, and additional research is required to fully address this interesting topic. It is also noteworthy that FoxO-mediated transcription is not the only mediator of the insulin and insulin-like growth factor-1 cascade, and that several factors might therefore be involved in the pathogenesis of dementia. As an example, insulin resistance and inflammation, observed in people with an excess of visceral adiposity, are also believed to contribute to metabolic deterioration of skeletal muscle, manifesting clinically as sarcopenia. M. E. Levine and E. M. Crimmins investigated the influence of insulin resistance and inflammation on the association between body composition and cognitive performance in older adults. The study included 1127 adults from the US National Health and Nutrition Examination Survey (NHANES 1999–2002) and showed that body composition does not predict cognitive functioning in adults aged 60–69 years, but, for adults aged 70 years and over, sarcopenia and obesity, either independently or concurrently, were associated with worse cognitive functioning (WAIS III, Digit Symbol substitution performance) relative to nonsarcopenic nonobese older adults. Cognitive functioning was lowest among the sarcopenic obese group, and sarcopenic obese people also showed the highest levels of inflammation. Moreover, insulin resistance accounted for a significant proportion of the relationship between cognitive performance and obesity, with or without sarcopenia. This is a novel, interesting, and important study on the association between sarcopenic obesity, insulin resistance, and cognitive functioning strengthened by the large sample size and suggesting that individuals who are sarcopenic obese have a lower cognitive ability than other subjects, and that this association might be partially explained by insulin resistance and inflammation. Aging seems also to be an important factor to be considered in order to see a significant effect. The study has, however, several limitations that the authors have acknowledged, including the lack of longitudinal data to evaluate whether insulin resistance precedes frailty and cognitive decline, the use of a single measure of cognitive functioning to study cognitive performance, and the fact that insulin resistance and inflammation were measured only one point in time. However, it provides preliminary interesting data for the design of longitudinal studies to better address this issue. Collectively, these three papers provide new insight into the role that obesity, metabolic syndrome and sarcopenia might play in dementia, by focusing on possible biological mechanisms or by providing novel and interesting preliminary data on humans.

 G. L. Bowman et al. analysed 36 subjects with mild-to-moderate AD investigating the correlation between dyslipidemia and blood-brain barrier (BBB) impairment. The CSF-to-serum ratio of albumin (CSF Albumin Index) ≥9 was considered as BBB impairment. The study revealed that dyslipidemia was frequent in AD subjects with BBB impairment. Furthermore, patients with BBB dysfunction showed significantly higher mean plasma triglyceride and lower HDL cholesterol levels with respect to those without BBB dysfunction. Overall, plasma triglycerides explained 22% of the variance in BBB integrity and remained significant after correcting for age, gender, *APOE-*ε*4* genotype, blood pressure, and statin use. This research paper adds to the growing literature on BBB dysfunction in AD by suggesting that dyslipidemia may have a detrimental role in maintaining BBB integrity in mild-to-moderate AD. The limit of this study is the very small sample size, and additional studies are required to demonstrate a causal link between dyslipidemia and BBB impairment. However, if replicated in other populations, these findings might gain clinical significance because dyslipidemia is treatable.

Another important issue is that of the difficulties associated with maintaining adequate nutrition in individuals with dementia. Nutrition and appetite decline with age, often accompanied by weight loss. This is particularly critical in advanced dementia where progressive feeding problems can become so severe that physicians and families must decide whether artificial nutrition and hydration is required. This important issue is discussed in the review article by G. A. Pivi and colleagues.

B vitamins and methyl-group homeostasis have received considerable attention in recent years, providing a basis for understanding the complex interplay between nutrition and epigenetic modifications of disease-related genes, including those that are involved in aging and in Alzheimer's disease. For example, experimental modification of methyl-group homeostasis through dietary deficiency and supplementation of choline and folate has been shown to exert profound effects on brain development, function, and aging [10–12], including epigenetic modification and/or aberrant expression of key AD genes [13]. In light of this attention, it has been turned to the potential impact of food folic acid fortification and nutritional status in human metabolic programming [14, 15]. 

Nicotinamide methylation is another potentially important mechanism that is theoretically susceptible to altered methylation potential, but one that is far less studied. Dietary impairment of this pathway might contribute to disturbed energy metabolism and cholinergic neurotransmission in dementia. A. C. Williams and colleagues draw attention to the role that this pathway may play in age-related cognitive impairment, by taking pellagra, as an example, a severe vitamin deficiency disease most commonly caused by a chronic lack of niacin (vitamin B3) in the diet, and leading to dementia, dermatitis, diarrhoea, and death. Niacin status is not well studied in relation to dementia risk, either alone or in relation to methyl-group metabolism although there are experimental animal data showing benefits of supplementation [16]. More research in this area is warranted. 

Overall, it is becoming clear that dietary factors play a fundamental role in brain health throughout the lifespan. Improving nutrition and dietary habits during early life and adulthood might therefore be an effective strategy to counteract age-related diseases. 

We hope that this special issue will contribute to our understanding of the complex interplay between nutrition and dementia and thereby advance our collective efforts to prevent, delay, and manage this debilitating disease.

We are extremely grateful to all the authors for their contributions that made possible to cover several timely topics in this special issue. 
